# Supplementary material for: Modification of Cotton Fabric with Molecularly Imprinted Polymer-Coated Carbon Dots as a Sensor for 17 α-methyltestosterone
Source: Molecules. 2022 Oct 26;27(21):7257. doi: 10.3390/molecules27217257 (PMC9658829; doi:10.3390/molecules27217257)
Supplement: Supplementary file 1 [file molecules-27-07257-s001.zip › molecules-1963047-supplementary.pdf]

Modification of Cotton Fabric with Molecularly Imprinted Polymer-Coated Carbon Dots as a Sensor for 17 α-methyltestosterone

Monyratanak Lim [1], Sudtida Pliankarom Thanasupsin [2] and Nisakorn Thongkon [1,*]

[1] Department of Chemistry, Faculty of Science, King Mongkut's University of Technology Thonburi, Bangkok 10140, Thailand;
[2] Chemistry for Green Society and Healthy Living Research Unit, Faculty of Science, King Mongkut's University of Technology Thonburi, Bangkok 10140, Thailand
* Correspondence: nisakorn.tho@kmutt.ac.th; Tel.: +66-61-854-15-91

Supplementary Data

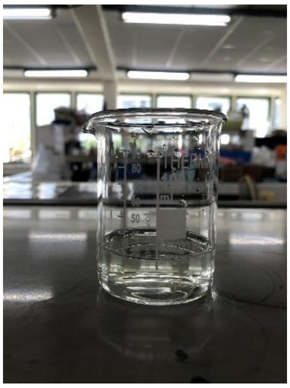

CA+H_2O 8 min

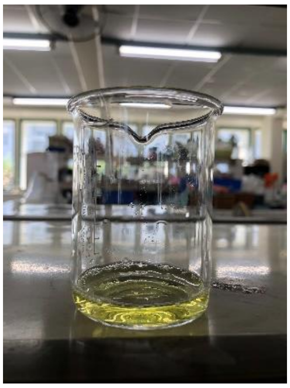

CA+EDA 5 min

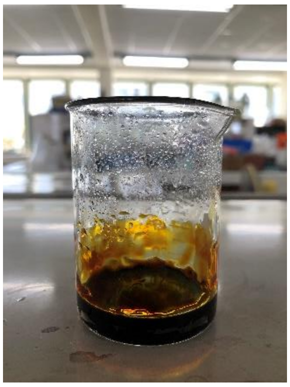

CA+EDA 8 min

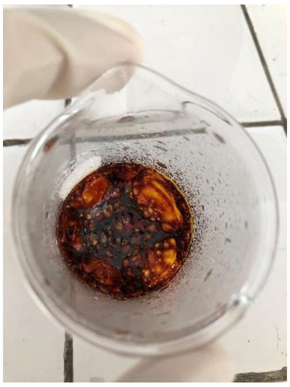

CA+EDA 9 min

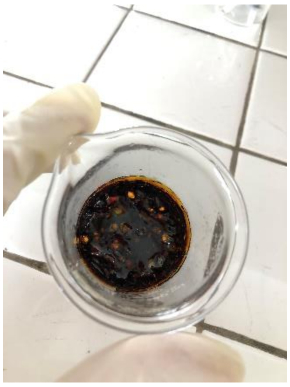

CA+EDA 10 min

Figure S1. Images of the obtained EDA-CDs with various exposure times at 300W.

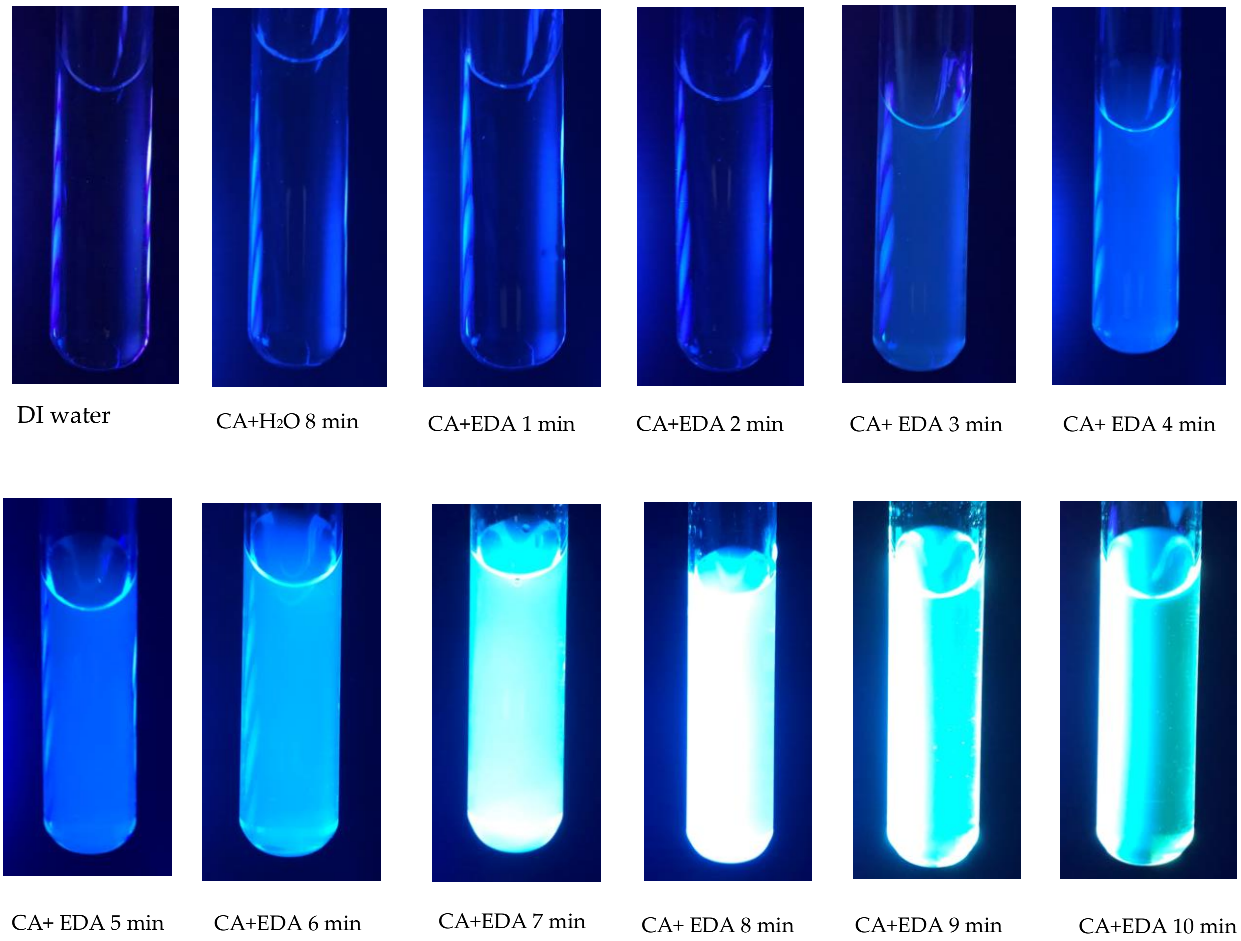

Figure S2. Images of carbon dots solution (1000 mg/L) with various exposure times from 1 to 10 min at 300 W under UVA radiation.

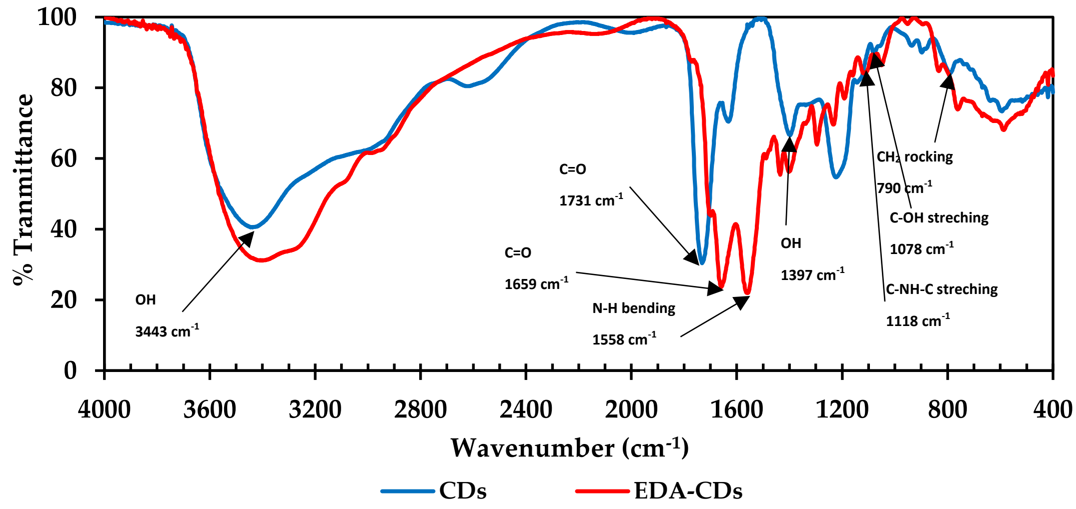

Figure S3. FTIR spectra of CDs (blue) andEDA-CDs (red) from synthesis using a microwave at 300 W for 8 min.

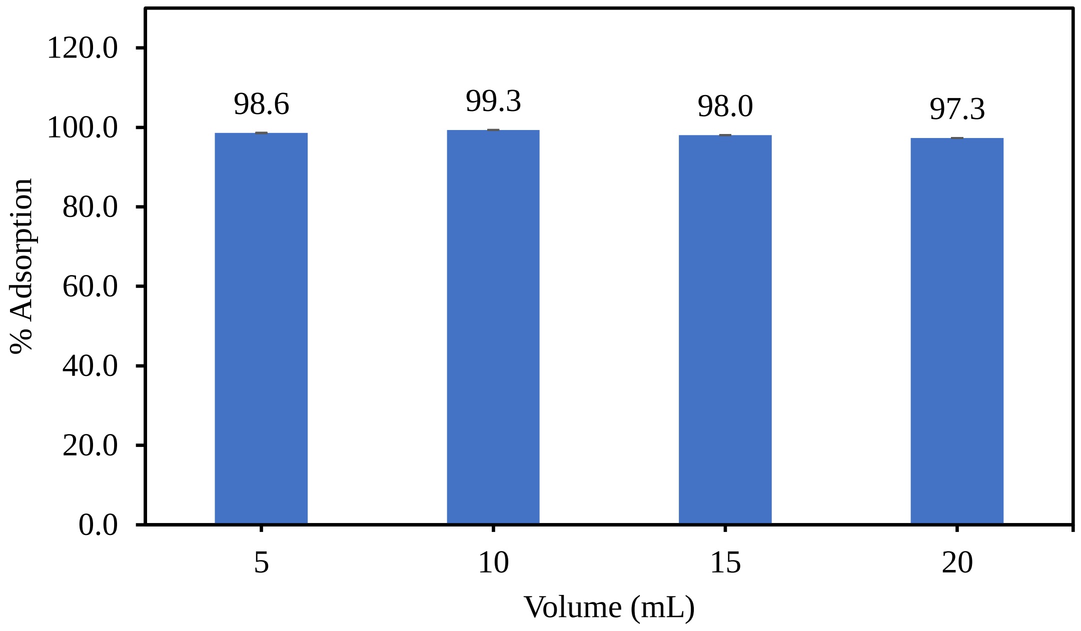

Figure S4. % Adsorption of the EDA-CDs grafted cotton fabric with various volumes of 1000 mg/L EDA-CDs for 10 min of sonication time.

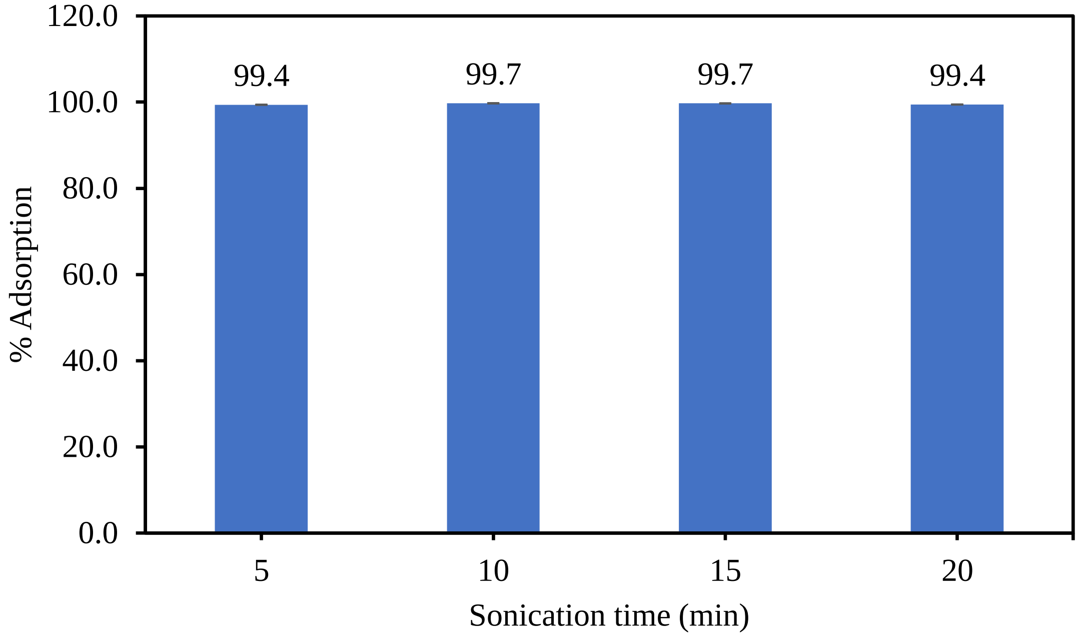

Figure S5. % Adsorption of the EDA-CDs grafted cotton fabric with various sonication times for 10 mL of 1000 mg/L EDA-CDs.

Table S1. Optimization for preparation of the EDA-CDs/CF.

Parameters	Gray Intensity (n=3)	Fluorescence Image
**Volume of the EDA-CDs solution (mL)**		
5	217±1.2	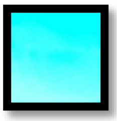
10	227±1.3	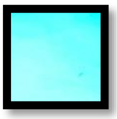
15	225±0.3	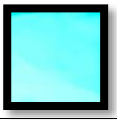
20	222±0.3	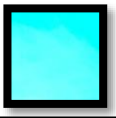
**Sonication time (min)**		
5	222±2.7	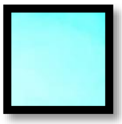
10	229±0.6	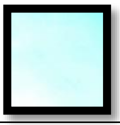
15	225±0.4	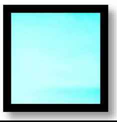
20	224±0.6	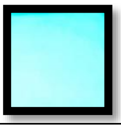

Table S2. Optimization for preparation of the MIPs/MT@EDA-CDs/CF.

**Parameters**					Gray intensity (n=3)	Fluorescence Image
MT concentration (mg/L)	MAA (μL)	EGDMA (μL)	AIBN (μL)	Polymerization time (hour)		
25	400	700	40	16	222 ± 5.6	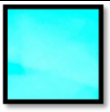
**50**					238 ± 4.5	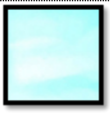
75					207 ± 2.4	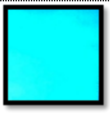
100					234 ± 2.9	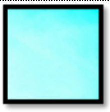
50	**300**	700	40	16	249 ± 2.9	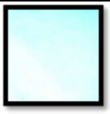
	400				245 ± 0.8	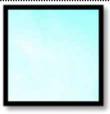
	500				239 ± 11.9	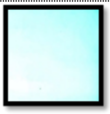
	600				239 ± 5.87	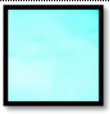
50	300	600	40	16	229 ± 2.3	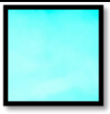
		**700**			248 ± 0.2	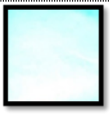
		800			245 ± 2.7	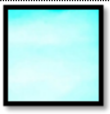
		900			240 ± 2.0	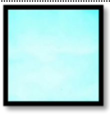

Table S2. Optimization for preparation of the MIPs/MT@EDA-CDs/CF (Continue).

Parameters					Gray intensity	Fluorescence Image
MT concentration (mg/L)	MAA (μL)	EGDMA (μL)	AIBN (μL)	Polymerization time (hour)		
50	300	700	30	16	225 ± 2.9	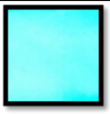
			40		245 ± 3.8	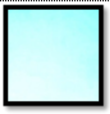
			**50**		250 ± 2.5	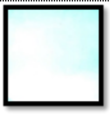
			60		246 ± 2.0	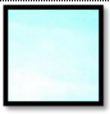
50	300	700	50	16	242 ± 0.7	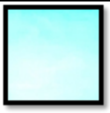
				18	248 ± 4.7	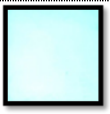
				20	249 ± 1.4	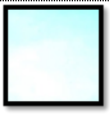
				**22**	251 ± 1.1	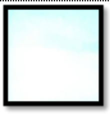

Table S3. Optimization of desorption solvent to extract MT from the MIPs/MT@EDA-CDs/CF.

**Extraction Solvent**	**Gray Intensity**	**Elution efficiency (%)**	**Fluorescence Image**
Control (NIP)	157 ± 0.8	-	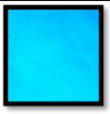
Water	170 ± 6.4	-	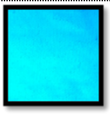
**EtOH: ACN; 1:4**	168 ± 0.1	98.3	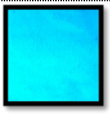
EtOH: ACN; 2:3	173± 1.8	96.3	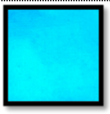
EtOH: ACN; 3:2	172± 0.9	93.8	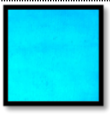
EtOH: ACN; 4:1	208± 3.3	82.1	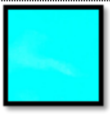

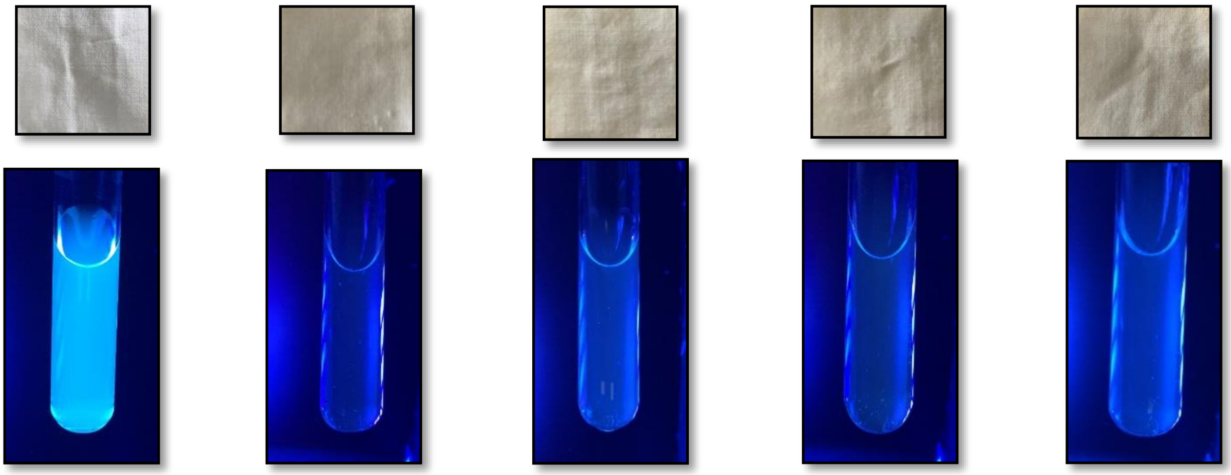

Water EtOH: ACN; 1:4 EtOH: ACN; 2:3 EtOH: ACN; 3:2 EtOH: ACN; 4:1

Figure S6. The original images of the MIPs@EDA-CDs/CF under normal light and the remaining solutions under UVA radiation.

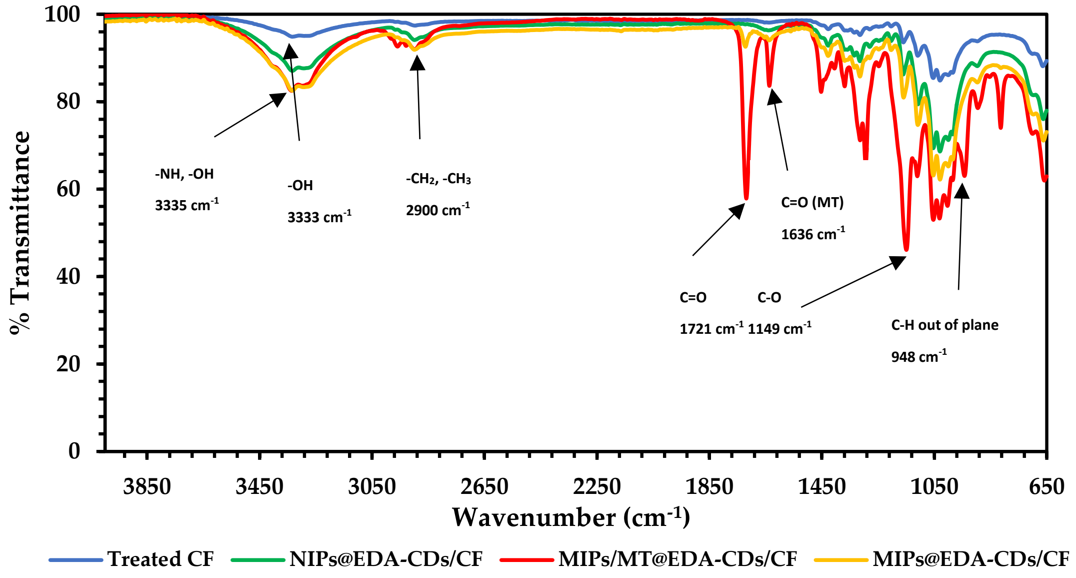

Figure S7. ATR-FTIR transmission spectra of treated cotton fabric, MIPs/MT@EDA-CDs/CF, NIPs@EDA-CDs/CF, MIP@EDA-CDs/CF

Table S4. RGB measurement of the MIPs/EDA-CDs/CF for MT detection at different concentration using smartphone-based image analysis.

Concentration (μg/L)	Color intensity			
	Gray	Red	Green	Blue
0	162	3	229	252
100	169	15	241	252
300	184	52	247	252
500	196	81	255	252
800	214	138	255	254
1000	227	175	254	251
